# Collaborative Efforts for Spinocerebellar Ataxia Research in the United States: CRC-SCA and READISCA

**DOI:** 10.3389/fneur.2020.00902

**Published:** 2020-08-26

**Authors:** Chih-Chun Lin, Tetsuo Ashizawa, Sheng-Han Kuo

**Affiliations:** ^1^Department of Neurology, College of Physicians and Surgeons, Columbia University, New York, NY, United States; ^2^Initiative for Columbia Ataxia and Tremor, Columbia University, New York, NY, United States; ^3^Department of Neurology, Houston Methodist Research Institute, Houston, TX, United States

**Keywords:** ataxia, cerebellum, network, consortium, spinocerebellar ataxia

## Abstract

Spinocerebellar ataxias are progressive neurodegenerative disorders primarily affecting the cerebellum. Although the first disease-causing gene was identified nearly 30 years ago, there is no known cure to date, and only a few options exist for symptomatic treatment, with modest effects. The recently developed tools in molecular biology, such as CRISPR/Cas9 and antisense oligonucleotides, can directly act on the disease mechanisms at the genomic or RNA level in disease models. In a nutshell, we are finally just one step away from clinical trials with therapies targeting the underlying genetic cause. However, we still face the challenges for rare neurodegenerative diseases: difficulty in obtaining a large cohort size for sufficient statistical power and the need for biomarkers and clinical outcome assessments (COA) with adequate sensitivity to reflect progression or treatment responses. To overcome these obstacles, ataxia experts form research networks for clinical trial readiness. In this review, we retrace our steps of the collaborative efforts among ataxia researchers in the United States over the years to study and treat these relentless disorders and the future directions of such research networks.

## Introduction

Spinocerebellar ataxias (SCAs) are a group of neurodegenerative disorders involving the cerebellum with an autosomal-dominant pattern of inheritance. SCAs are monogenetic disorders with a high disease penetrance and defined clinical presentations with the core feature of cerebellar ataxia; therefore, SCAs can serve as disease models for novel disease-specific therapeutic approaches, such as gene therapies or antisense oligonucleotides (ASOs).

The pooled prevalence of hereditary ataxia is ~2.7–38.35 per 100,000 (1–3); therefore, SCAs are considered orphan diseases. The major research challenges for orphan diseases are patient recruitment, development of reliable and responsive disease-specific clinical outcome assessment (COA) measures, collection of biosamples for biomarker discovery, uniform acquisition of brain imaging data, and the understanding of natural history. Addressing these challenges through collaborative research by a network of investigators specializing in such diseases is a powerful approach to establish clinical trial readiness.

The goal of this article was to review the history, the current state, and the future perspectives of an ataxia research network in the United States. Through the collaboration between the ataxia research networks and industries, several clinical trials for SCAs have been launched. These milestones for SCA research bring hope to SCA patients and their family members.

## History of SCA Research in the United States

**Figure 1:**
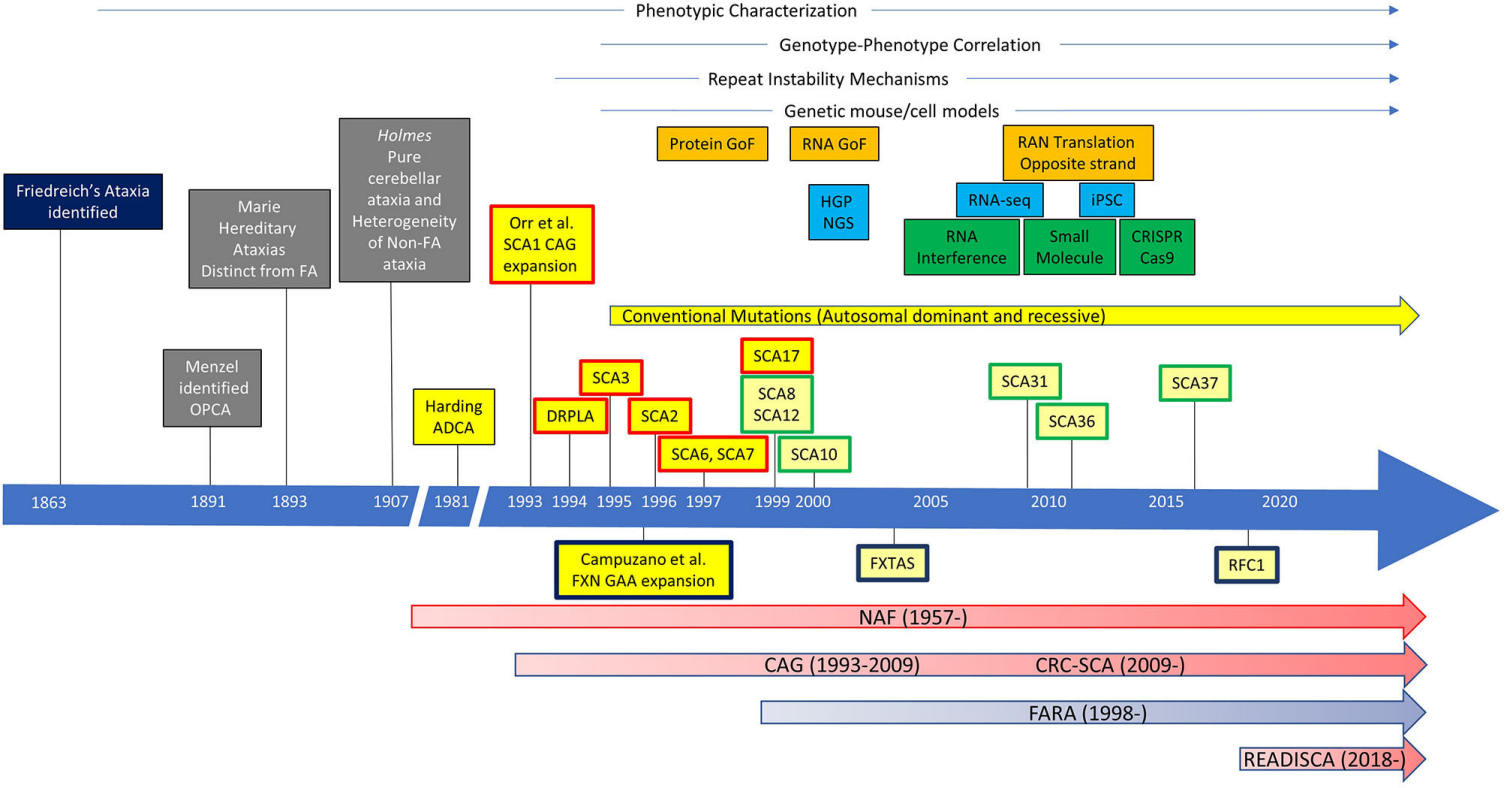
The history of ataxia research. Ataxias caused by repeat expansions were labeled, with autosomal-dominant ataxias above and autosomal-recessive ataxias below the timeline. Spinocerebellar ataxias (SCAs) with repeat expansion in the coding region are in boxes with a red outline. SCAs with repeat expansion in the non-coding region are in boxes with a green outline. GoF, gain of function; HGP, Human Genome Project; NGS, next-generation sequencing; RNA-seq, RNA sequencing; iPSC, induced pluripotent stem cells; OPCA, olivopontocerebellar atrophy; FA, Friedreich's ataxia; ADCA autosomal-dominant cerebellar ataxia; DRPLA, dentatorubral pallidoluysian atrophy; FXN, frataxin; FXTAS, Fragile X-associated tremor/ataxia syndrome; RFC1, replication factor C subunit 1; NAF, National Ataxia Foundation; CAG, Cooperative Ataxia Group; CRC-SCA, Clinical Research Consortium for Spinocerebellar Ataxias/Clinical Research Consortium for Studies of Cerebellar Ataxias; FARA, Friedreich's Ataxia Research Alliance.

The history of collaborative ataxia research could be dated back before the genetic discovery to define each SCA subtype. In 1957, the National Ataxia Foundation (NAF) was established, marking the prelude of organized collaboration for clinical studies of ataxia (Figure 1). In 1975, the first joint meeting for ataxia research took place in Minneapolis, chaired by Dr. Lawrence Schut, to achieve the goal of promoting research collaboration between clinical, genetic, and basic science research of ataxia. Subsequently, the first International Symposium on Inherited Ataxias was held in 1977 in Los Angeles, which drew almost 100 researchers representing five countries. These meetings, which were the first of their kind focusing solely on ataxia, led to collaborative efforts between investigators.

In 1993, a group of investigators led by Huda Zoghbi and Harry Orr identified a heterozygous expansion of CAG repeat that encodes a polyglutamine (polyQ) tract in a novel gene, *ATXN1*, in a family with an autosomal-dominant cerebellar ataxia, now known as spinocerebellar ataxia type 1 (SCA1) (4). This pivotal work triggered a “gold rush” in the discoveries of new SCAs, particularly those caused by polyQ expansions in the coding region, including dentatorubral pallidoluysian atrophy (DRPLA), SCA2, SCA3, SCA6, SCA7, and SCA17. In addition, SCAs caused by repeat expansions in the non-coding regions (e.g., SCA8, SCA10, SCA12, SCA31, SCA36, and SCA37) and other traditional mutations have been identified, which are still growing in number today (up to SCA48) (5, 6). There has been no more coding-region polyQ expansion SCAs identified after 1999, but polyQ SCAs are collectively the most common among all SCAs.

Although the discovery of polyQ expansion mutation has given rise to a strong hope for the development of rational therapeutic interventions, successful clinical trials have not been forthcoming for efficacious treatments. However, understanding of the pathogenic molecular pathways triggered by the polyQ expansion has been advancing at an accelerating pace for the past 10 years, and several promising drug development programs have emerged. Among them, RNA silencing is attracting strong attention by academic investigators, the pharmaceutical industry, and patient support groups. While preclinical studies of ASOs, microRNAs (miRNAs), and other RNA silencing technologies are progressing nicely, clinical trial readiness remains suboptimal.

However, the first effort for clinical trial readiness was not successfully put together until 1997, when the Ataxia Neuropharmacology Committee of the World Federation of Neurology introduced the International Cooperative Ataxia Rating Scale (ICARS) (7). Although clinical ataxia researchers started using ICARS extensively, ICARS was soon found to be cumbersome, with redundancy in the subscale structure and concerns about its usefulness for future interventional trials. Meanwhile, a NAF-sponsored group of US clinical investigators formed the first clinical ataxia consortium, the Cooperative Ataxia Group (CAG). The CAG had constructed and validated the Friedreich's Ataxia Rating Scale (FARS) (8) and was intensely revising ICARS to address this problem when European investigators published the Scale and Assessment for Rating of Ataxia (SARA) in 2006 (9). Because SARA closely resembled what the CAG was drafting as a new ataxia scale, the Unified Ataxia Disease Rating Scale (UADRS), the CAG made a decision to abandon their own efforts. This was a critical decision that later enabled unifying the clinical researchers of ataxia across the Atlantic. While the Europeans launched the European Integrated Project on Spinocerebellar Ataxias (EUROSCA) and Prospective Study of Individuals at Risk for Spinocerebellar Ataxia (RISCA) (10, 11), the CAG started conducting the first multicenter natural history study of SCAs in the United States (12). The CAG was registered as one of the National Institutes of Health (NIH) Rare Diseases Clinical Research Consortia and acquired a new designation, “Clinical Research Consortium for Spinocerebellar Ataxias (CRC-SCA).” Upon conclusion of the 2-years natural history study of SCA1, SCA2, SCA3, and SCA6, the CRC-SCA changed the acronym for SCA to “Studies of Cerebellar Ataxia” under NAF sponsorship. In 2017, the CRC-SCA initiated an NIH-funded 5-years project, “Clinical Trial Readiness for SCA1 and SCA3 (READISCA).” READISCA (NCT03487367) is the first US–European collaborative SCA project and focuses on premanifest and early-stage subjects of SCA1 and SCA3 mutation carriers. This 5-years longitudinal study uses SARA as the primary COA measure with corresponding magnetic resonance spectroscopic (MRS) and MR imaging (MRI) biomarkers, collects biofluid samples, and assesses trial designs by simulations using the clinical and biomarker data.

## CRC-SCA

**Figure 2:**
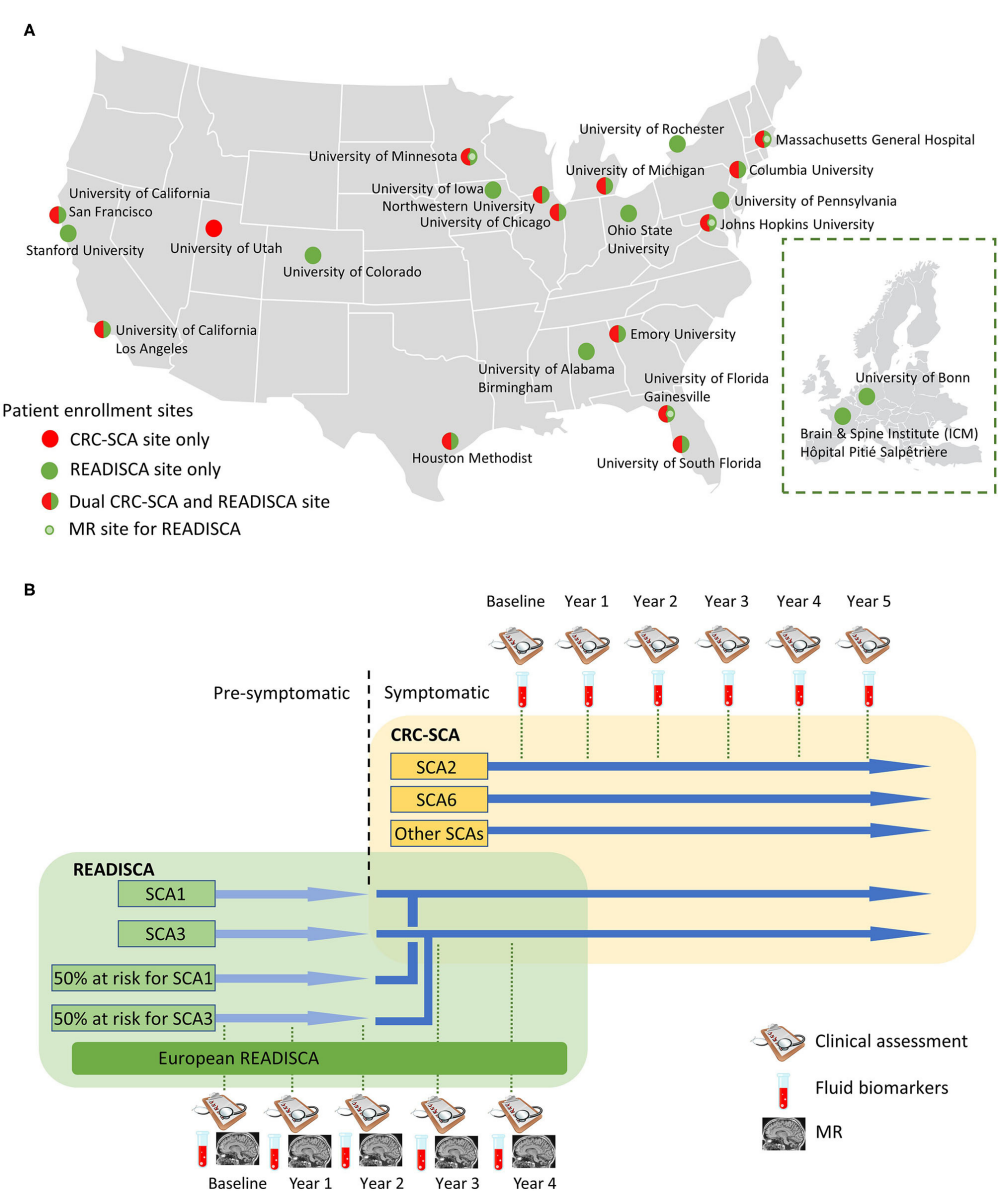
Site and study overview of the Clinical Research Consortium for Spinocerebellar Ataxias (CRC-SCA) and Clinical Trial Readiness for SCA1 and SCA3 (READISCA). **(A)** Patient enrollment sites for CRCSCA and READISCA. Inset: participating sites of READISCA in Europe. **(B)** Study design of CRCSCA and READISCA.

The natural history study of CRC-SCA (NCT03487367) originally focused on the various COAs and genetic modifiers for SCA1, SCA2, SCA3, and SCA6 and later expanded to other repeat expansion SCAs, including SCA7, SCA8, and SCA10. This ongoing natural history study currently has 14 patient enrollment sites (Figure 2A) to investigate the clinical characteristics and progression of genetically confirmed, symptomatic SCA patients (Figure 2B). The natural history records the longitudinal progression data of ataxia severity (measured by SARA), depressive symptoms associated with ataxia (measured by Patient Health Questionnaire-9, PHQ-9), and functional capacity (measured by the Unified Huntington's Disease Rating Scale Part IV, UHDRS-IV). Various extracerebellar features, such as dystonia or tremor, are captured by the Inventory of Non-Ataxia Signs (INAS) (13). After the development of the Cerebellar Cognitive Affective Syndrome Scale (CCAS) in 2018 (14), this scale is also included to comprehensively assess SCA patients' cognitive function.

As the result of the CRC-SCA natural history study, we found that the rates of disease progression of SCA1, SCA2, SCA3, and SCA6 (annual increase in SARA by 1.61, 0.71, 0.65, and 0.87 points, respectively) (12) are consistent with those in EUROSCA (15). In addition, we found that the severity of depressive symptoms also tracks along with ataxia progression (16), while dystonia and tremor could be prominent features of SCA patients in a subtype-specific manner (17–19). Another important piece of information from this cohort is that we found that the occurrence of cardiovascular risk factors is quite low (20), which will have implications in assessing the vulnerability to side effects for novel therapies.

In addition to the clinical data, blood samples for DNA extraction were sent to the University of Utah to determine the repeat expansions in various genes to further investigate the consequences of repeat interactions in SCAs (21). Specifically, clinical presentations of tremor and dystonia could be influenced by the repeat expansions outside of the pathological SCA allele (17, 18). We also recently identified that the pathological repeat expansions of *C9orf72* occur in a small subset of SCA patients, and the intermediate repeat expansions of *C9orf72* can be a genetic modifier for depressive symptoms (22), further underscoring the importance of repeat interactions. Another discovery related to genetic modifiers is that ethnicity can play a role in SCA disease progression (23).

A critical aspect of the ongoing CRC-SCA is the recent expansion to collect blood and cerebrospinal fluid (CSF) for biomarker discovery for symptomatic patients. These fluid samples will be collected longitudinally; therefore, we will have the capacity to discover markers that track disease progression.

## READISCA

READISCA, an extensive NIH-funded multinational clinical trial readiness study, was initiated in 2017 and currently has 20 US and two European sites for clinical assessment and biofluid collection, and among these, four sites are performing neuroimaging studies (Figure 2A). A component of READISCA overlaps with CRC-SCA to study early symptomatic SCA1 and SCA3 patients with SARA ≤ 9.5. However, different from CRC-SCA, which enrolls patients from all stages of diseases, READISCA studies the early stage of diseases to plan for future clinical trials studying disease-modifying therapies. READISCA thus includes pre-symptomatic SCA1 and SCA3 patients and also 50% at-risk patients who do not exhibit ataxia symptoms and who do not know their genetic status. Patients in READISCA are anticipated to enter clinical trials in the next 5 years. The reasons for choosing SCA1 and SCA3 are that SCA1 is the fastest progressing polyQ SCA (12) while SCA3 represents the most common SCA in most regions of the world (24).

In READISCA, there is an emphasis on MR as an imaging biomarker for SCA1 and SCA3. While conventional structural MRI has been the standard of care to monitor the characteristic cerebellar and brainstem atrophy in patients with SCA1 and SCA3, this technique is limited by the lack of sensitivity in the premanifest stages (25, 26). On the other hand, the use of multimodal MRI with a combination of volumetry, voxel-based morphometry, and diffusion tensor imaging, along with MRS and resting-state functional MRI, can serve as sensitive imaging biomarkers for presymptomatic and early stages of SCAs. Specifically, MRS has been demonstrated to measure neurochemical abnormalities in the presymptomatic stages (27) and the treatment effects of transgenic SCA1 mouse models with high sensitivity and specificity (28). For SCA1 and SCA3, volumetric analysis showed that the rate of cerebellum volume decrement correlated with the rate of SARA increase (29, 30). Similarly, diffusion tensor imaging revealed that metrics, such as fraction anisotropy and mean diffusivity in the brainstem and cerebellum can reflect the change in SARA (31, 32) and ICARS (33). The validation of the use of MR techniques in this patient population is highly relevant and will have implications for disease-modifying therapies in the early stages. Similar to CRC-SCA, READISCA also prospectively collects patient blood and CSF samples for biomarker discovery.

Another unique component of READISCA is that it includes two sites in Europe, one in Paris, France, and the other in Bonn, Germany (Figure 2B). The data will eventually be compiled and analyzed together with sites in the United States, which can serve as the basis for trans-Atlantic collaboration.

## Centralized Data and Biospecimen Storage

Since CRC-SCA and READISCA have many shared components in terms of investigators, patients and their family members, COAs, and biospecimens, these two ataxia networks also share a centralized data storage to facilitate the integration and analysis of the data throughout the disease courses. The storage of data and biospecimens is monitored by the leadership of the respective ataxia networks. Clinical data are stored at the University of South Florida Health Informatics Institute, whereas imaging data are stored at the University of Minnesota. Biospecimens, including blood and CSF, are sent to BioSEND, which is the National Institute of Neurological Disorders and Stroke (NINDS) biomarker repository at Indiana University. These biospecimens are made available to academic and industry researchers through committee review and approval by the Biospecimen Resource Access Committee. Finally, the blood DNA samples are stored and analyzed at the University of Utah. All these facilities have extensive experience in performing clinical studies and serve as key foundations for CRC-SCA and READISCA.

## Challenges for CRC-SCA and Readisca

READISCA is designed to recruit early-stage patients, pre-symptomatic patients, and subjects with unknown genetic status but with affected first-degree relative(s). The goal of this non-treatment study may not meet the expectations of early-stage and pre-symptomatic patients who are seeking for a cure or a disease-modifying treatment. Similar challenges also occur in CRC-SCA. The other “challenge” is the development of new clinical trials studying therapeutic agents. Many patients might choose to participate in these clinical trials instead of continuing in the natural history study of CRC-SCA and READISCA. This particular challenge is in fact the original goal of CRC-SCA and READISCA, to eventually transition SCA patients into clinical trials.

CRC-SCA and READISCA were also challenged by difficulties in patient recruitment, meeting the expectations of different funding agencies, and the recent changes in the laws regulating personal information sharing. These challenges may also be encountered by clinical trials studying other rare diseases. The lessons learned below may benefit future clinical trials.

An intrinsic challenge for both CRC-SCA and READISCA is recruiting a sufficient number of patients. This was partly overcome by the active participation of patients and their families, who are highly motivated to participate in the natural history study with the hope of finding therapies for SCAs.

The funding sources for CRC-SCA and READISCA are from NAF and NINDS, respectively. Sustained funding from the industry is needed in the future. While many pharmaceutical companies are interested in supporting these ataxia networks, each industry partner might want to support different components of the study. For example, CSF biospecimen will have profound implication to test for target engagement of ASOs for disease-modifying therapies, and physiological measures may be relevant for monitoring the effects of ion channel modulators aimed at symptomatic treatments for ataxia. Organizing the diverse interests among individual pharmaceutical companies to support the ataxia research networks will be one important challenge. Along this line, the other consideration is the data sharing policy in different companies.

In May 2018, the General Data Protection Regulation (GDPR), a data protection law, was enforced across all European Union countries to set the boundaries and regulations for acquiring, processing, and storing personal information (34). GDPR is also applicable outside of Europe, as long as the personal information being processed belongs to someone who is physically located in the European Union. As READISCA proposed to merge the databases of SCA patients from the United States and Europe, it needs to be GDPR-compliant. While the Health Insurance Portability and Accountability Act and the Genetic Information Non-discrimination Act are less stringent equivalents to GDPR in the United States, several states have followed the steps of the European Union to legislate their own versions, such as the California Consumer Privacy Act (35). The strict GDPR laws clash with the NIH policy of widely sharing data and resources obtained with the NIH grants. This discordance resulted in countless sessions with attorneys on both sides of the Atlantic, with a substantial delay in the READISCA enrollment. READISCA and CRC-SCA will need to continue to adapt as additional regulations from different states emerge to eventually merge the data with the European counterpart.

## Future Perspectives

ASOs have been successfully applied for the treatment of spinal muscular atrophy (36) and demonstrated promising results in Huntington's disease (37). These results have shown promise for ASOs to treat monogenetic neurological disorders. Therefore, ASOs and other RNA silencing molecules have been developed in SCA preclinical models and have been shown to mitigate motor symptoms in mouse models of SCA1 (38, 39), SCA2 (40), and SCA3 (41) as well as prevent blindness in a mouse model of SCA7 (42). While ASOs, virus-mediated gene therapies, and other molecular interventions targeting pathways specific for each SCA (43) have been developed in an unprecedented speed at the preclinical stages, READISCA and CRC-SCA are pivotal SCA networks to prepare for the clinical trials by (1) recruiting SCA patients, (2) developing and validating COAs, and (3) discovering imaging and fluid biomarkers. In addition, gene-editing technologies, such as zinc finger nuclease and CRISPR/Cas9, with their ability to precisely edit the genome, have brought hope to SCA patients to manipulate the disease at the genomic level. In particular, CRISPR/Cas9 has been successfully applied to delete the expanded CAG repeats in induced pluripotent stem cells (44). Another innovative therapy on the horizon is mesenchymal stem cell infusion (45). These new therapies can be developed on the established platforms of CRC-SCA and READISCA.

READISCA can be viewed as the first step in preparing for foreseeable clinical trials for disease-modifying therapies. While READISCA combines the forces of the United States and Europe, future efforts are needed to strengthen global collaboration. SCAs are a group of rare diseases, and only international cooperation can achieve sufficient sample sizes to reach enough power. Furthermore, certain types of SCAs may have high incidences regionally, for example, SCA1 in Poland, Russia, South Africa, Serbia, Italy, and India; SCA2 in Cuba, Mexico, Korea, India, Italy, and Spain; SCA3 in Portugal, Brazil, China, Netherlands, Germany, Japan, and Taiwan; and SCA8 in Finland (6, 46–49). An international task force with shared data will also be important to investigate how the ethnic, genetic, and/or environmental factors influence monogenetic disorders, such as SCAs. Finally, it is critical to standardize the COAs so the results obtained from one study can be compared with the other. Although SARA is the most extensively used and well-validated COA today, further modifications to improve SARA's responsiveness are ongoing. To further strengthen the international collaborations for these important goals, several international networks for ataxia research have recently been established. The Pan-American Hereditary Ataxia Network aims to facilitate the communications between Latin American countries and the United States. At the same time, the SCA Global initiative was established to enhance collaboration between researchers from the United States, Asia, and Europe. The SCA Global initiative will include not only the data from CRC-SCA but also those from EUROSCA, RISCA, and the Spastic Paraplegia and Ataxia Network. These strong networks for clinical SCA research will be the hope in bringing the new therapies for SCAs to reality.

## Author Contributions

C-CL, S-HK, and TA together wrote and contributed substantially to the manuscript as well as providing critical comments to the content. All authors contributed to the article and approved the submitted version.

## Conflict of Interest

The authors declare that the research was conducted in the absence of any commercial or financial relationships that could be construed as a potential conflict of interest.
